# A Survey of Deep Learning Based NOMA: State of the Art, Key Aspects, Open Challenges and Future Trends

**DOI:** 10.3390/s23062946

**Published:** 2023-03-08

**Authors:** Syed Agha Hassnain Mohsan, Yanlong Li, Alexey V. Shvetsov, José Varela-Aldás, Samih M. Mostafa, Abdelrahman Elfikky

**Affiliations:** 1 Optical Communications Laboratory, Ocean College, Zhejiang University, Zheda Road 1, Zhoushan 316021, China; 2 Ministry of Education Key Laboratory of Cognitive Radio and Information Processing, Guilin University of Electronic Technology, Guilin 541004, China; 3 Department of Smart Technologies, Moscow Polytechnic University, Moscow 107023, Russia; 4 Faculty of Transport Technologies, North-Eastern Federal University, Yakutsk 677000, Russia; 5 Centro de Investigaciones de Ciencias Humanas y de la Educación (CICHE), Universidad Indoamérica, Ambato 180103, Ecuador; 6 Computer Science Department, Faculty of Computers and Information, South Valley University, Qena 83523, Egypt; 7 College of Engineering, Arab Academy for Science, Technology and Maritime Transport, Alexandria 21500, Egypt

**Keywords:** NOMA, Successive Interference Cancellation (SIC), Channel State Information (CSI), spectral efficiency, massive connectivity, deep learning, resource allocation

## Abstract

Non-Orthogonal Multiple Access (NOMA) has become a promising evolution with the emergence of fifth-generation (5G) and Beyond-5G (B5G) rollouts. The potentials of NOMA are to increase the number of users, the system’s capacity, massive connectivity, and enhance the spectrum and energy efficiency in future communication scenarios. However, the practical deployment of NOMA is hindered by the inflexibility caused by the offline design paradigm and non-unified signal processing approaches of different NOMA schemes. The recent innovations and breakthroughs in deep learning (DL) methods have paved the way to adequately address these challenges. The DL-based NOMA can break these fundamental limits of conventional NOMA in several aspects, including throughput, bit-error-rate (BER), low latency, task scheduling, resource allocation, user pairing and other better performance characteristics. This article aims to provide firsthand knowledge of the prominence of NOMA and DL and surveys several DL-enabled NOMA systems. This study emphasizes Successive Interference Cancellation (SIC), Channel State Information (CSI), impulse noise (IN), channel estimation, power allocation, resource allocation, user fairness and transceiver design, and a few other parameters as key performance indicators of NOMA systems. In addition, we outline the integration of DL-based NOMA with several emerging technologies such as intelligent reflecting surfaces (IRS), mobile edge computing (MEC), simultaneous wireless and information power transfer (SWIPT), Orthogonal Frequency Division Multiplexing (OFDM), and multiple-input and multiple-output (MIMO). This study also highlights diverse, significant technical hindrances in DL-based NOMA systems. Finally, we identify some future research directions to shed light on paramount developments needed in existing systems as a probable to invigorate further contributions for DL-based NOMA system.

## 1. Introduction

Cellular networks are becoming denser and more sophisticated because of the expansion in demand for wireless services with extremely fast speeds and low latency. One of the main problems cellular network operators have is developing and managing networks with an extensive number of components and characteristics. Consequently, self-organizing networks (SONs) have emerged as crucial components in managing wireless cellular networks [1]. By limiting human involvement in a network through various capabilities, such as self-healing, self-optimization, and self-configuration, SON technology seeks to lower capital and operating costs [2]. A significant use case for self-healing is the control of cell outages. It is applicable to base stations (BSs) that are no longer able to provide services to customers inside their zone, creating a coverage gap in the network [3]. To enhance coverage and capacity, cellular networks are using many accessing techniques like frequency division multiple access (FDMA), time division multiple access (TDMA), code division multiple access (CDMA) and orthogonal frequency division multiple access (OFDMA). These accessing techniques use the concept of orthogonality to reduce the interference between the users, but it also reduces the number of users multiplexed to access the spectrum. To increase the spectral efficiency (SE) of networks and the throughput of cell-edge users by enabling more users as compared to the available orthogonal resources, non-orthogonal multiple access (NOMA) has emerged as a promising technique in 5G networks [4]. NOMA can be integrated with MIMO, cognitive radio (CR), HetNets, milli-meter waves, mobile edge computing (MEC), visible light communication (VLC), vehicle communication, etc. This will provide high spectral efficiency, data rates and massive connectivity and decreases inter-cell interference and intra-cell interference. Initially, NOMA was used with single cells to improve spectral efficiency. The spectral efficiency is increased by increasing the multiplexed users accessing the single channel with different channel gains. NOMA may be an excellent choice for cell outage compensation due to its capacity to improve performance for cell edge users. In this case, the cell edge users that are participating in the compensation procedure are the users who are experiencing an outage (the unsuccessful users). In [5], the authors attempted the first effort to represent the dual issue of unsuccessful user association and power control quantitatively in a NOMA-based cell outage compensation system.

NOMA is divided into the power domain NOMA (PDNOMA) and code domain NOMA (CDNOMA). In PDNOMA, the multiplexing is based on the transmitted power and in CDNOMA, multiplexing is based on code. Along with CDNOMA and PDNOMA, there were other NOMA techniques like signature NOMA (S-NOMA) and compressing-based NOMA (CS-NOMA). In a recent study, the authors investigate the power domain NOMA [4], which divides users into two groups: those close to the network infrastructure, who receive low transmission powers, and those further away, who receive higher transmission powers. It may provide features like fast throughput and minimal lag time in communication, which are necessary for meeting the criteria. The usual successive interference cancellation (SIC) [6] decoding approach is used at the receiver in a NOMA-based system to ensure the system meets the criteria. Multiple user devices increase the complexity of the wireless system in areas such as low-latency real-time transmission, secrecy rate maximization, resource allocation, and signal identification. Cooperative communication has been introduced in NOMA, which refers to the employment of a relay to improve the system’s capacity to serve many customers and to increase the transmission area. There are two varieties of cooperative NOMA communication: user-assisted and relay-assisted [7,8]. In relay-aided transmission, an extra relay aids in communication between users; in this scenario, a near user helps a remote user to send data. A relay either decodes the message or transmits it to the recipient as is or amplifies it and sends it on to the recipient as a larger version [9].

**Figure 1:**
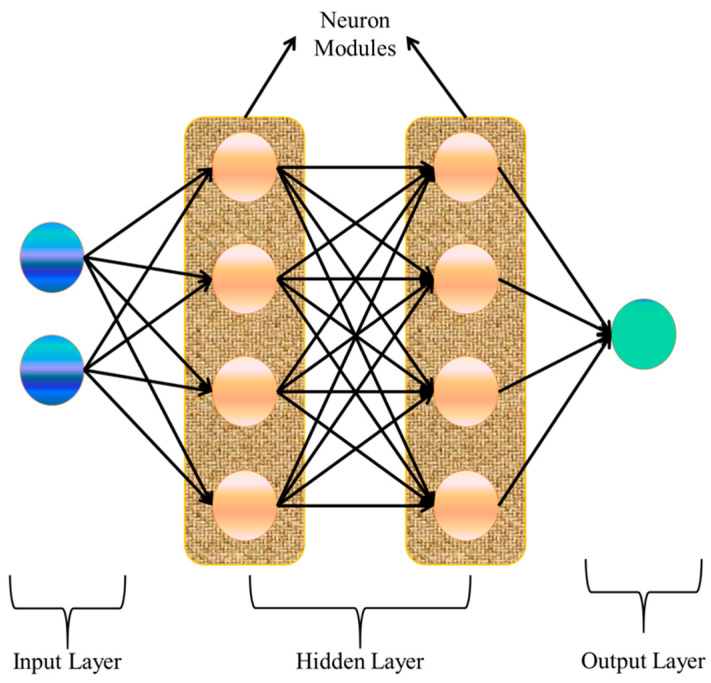
Deep neural network general framework.

Recently, deep learning-based NOMA systems have been utilized in several application scenarios. The field of study known as “deep learning” (DL) allows a system to learn and improve via exposure to data rather than through predetermined rules. Deep learning is a subset of machine learning. It has distinct advantages over traditional machine learning methods, such as being capable of working on huge volumes of data available for analysis purposes from complex networks owing to the growth of network sizes and usages. End-to-end classification solutions are conceivable from these metadata profiles with processing powers of graphic processing units (GPUs). A well-trained system can make sense of whatever data it is fed, extracting relevant information, and using that knowledge to identify and address issues. We may classify DL under three broad categories: reinforcement learning (RL), supervised, and unsupervised [10]. In supervised learning, a system learns how to make decisions based on examples that have already been categorized. Classification and regression are two applications that can benefit from supervised learning. Unsupervised learning permits a machine to carry out judgments considering unlabeled data and uncovers latent structure in an input. Association and clustering issues are common applications of algorithms based on unsupervised learning. Reinforcement learning describes a system’s ability to acquire skills via repeated practice. As it learns from its surroundings, reinforcement learning doesn’t need any input data to function. Reinforcement learning allows for the fully automatic detection and categorization of signals. Neural networks are used to implement DL algorithms [11]. The hidden layer, input layer, and output layer are the three components of a simple neural network, as shown in Figure 1. Deep neural networks (DNN) are so named because they have several hidden layers between the input and output levels. Neurons serve as the central processing units for each layer of the network. Recurrent neural networks (RNN) and convolutional neural networks (CNN) are two types of DNNs that differ in how they process inputs and generate outputs. In [11], the authors outline the steps to investigate these questions about DL in a NOMA-based system.

### 1.1. Background

Demanding applications such as virtual reality (VR), internet gaming, and high-definition (HD) films have contributed to a burgeoning information explosion [12] during the past decade. The advent of 5G has posed new difficulties in the areas of extensive connection, energy efficiency, peak data throughput, reduced latency, ultra-reliability, and spectral efficiency [13]. The rapid expansion of the Internet of Things (IoT)-based massively heterogeneous networks has necessitated incorporating a number of notable difficulties into 5G technology. Major obstacles to adopting modern multiple access systems now exist due to the uplink or downlink transfer of substantial user data between different networks. NOMA, an intriguing and potential solution for 5G networks, has attracted enormous attention recently [14] as a result of the aforementioned difficulties. Industry and academics alike have recognized NOMA as a promising trend and technology for meeting the varied requirements of 5G. Next-generation mobile or wireless networks rely on this crucial enabling technology to meet the varied demands of users for redundancy, speed, fairness, throughput, and connection. Through signal superposition, NOMA can serve an arbitrary number of users in each resource block.

Additionally, they may allocate power resources to nodes with poor channel characteristics to improve throughput. Compared to traditional multiple access (MA) methods, NOMA makes better use of available resources [15,16]. Unlike NOMA, each orthogonal resource unit in conventional MA techniques serves a single user. This has an adverse effect on the system’s overall throughput and spectrum effectiveness. When NOMA is used in these circumstances, it assures that not only users with poor channel characteristics are supplied, but also users with better channel conditions can consume a similar amount of bandwidth as the weak user. The multiuser signals in NOMA are broadcast to the end users after being multiplied in the transmission part through superposition coding (SC) at various power levels based on nonorthogonal symmetry.

Compared to consumers with better channel conditions, those with poorer channel conditions often receive more power. Therefore, having the proper channel state information (CSI) to transmit data has become more important in NOMA. With a strong channel gain, users may readily retrieve the signals gathered in the receiver. Users that experience weak channel capability mistake other signals for interference, which significantly lowers spectral efficiency. With NOMA, in the absence of a guard period and no signal interference, this issue may be solved. NOMA improves user fairness and provides great performance [17].

In contrast to fourth-generation (4G) networks, NOMA in 5G systems is primarily employed to enable larger user density and achieve high spectrum efficiency and low latency [18]. Significantly higher data speeds, increased system capacity, enormous numbers of mobile device connections, decreased latency and decreased power consumption may all be supported by 5G. A wide variety of data transmission entities with various data rates and latency demands make up 5G-enabled systems, including IoT systems. In 5G networks, where several users depend on the same resources, NOMA is employed. In the context of high spectral efficiency and dependable connection among multiple data transmission entities, NOMA in 5G systems is anticipated to satisfy the desiderata of 5G communication systems. Howbeit, NOMA systems have specific drawbacks, including high computational complexity, difficult designs, and issues with resource allocation.

**Figure 2:**
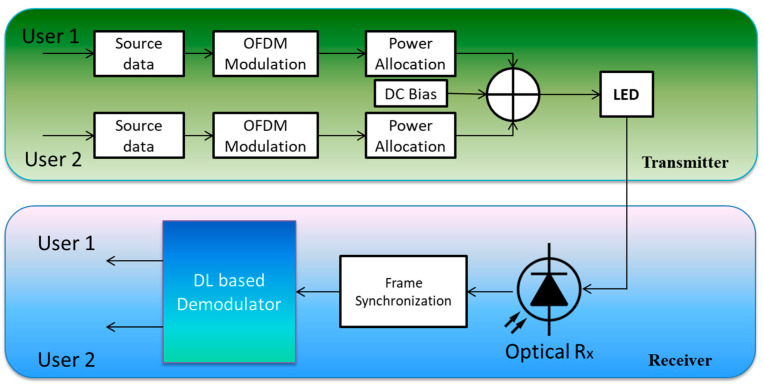
An illustration of DL-based NOMA-VLC system.

Furthermore, to perform consecutive interference nullification at the receiver side, NOMA systems also need flawless CSI. Perfect consecutive interference nullification is critical for improving NOMA performance. Without understanding the correct CSI at the system’s transmitter, it is extremely complex to build a successful power allocation (PA) technique. However, it might be challenging to obtain a near-perfect or flawless CSI. Deep learning (DL) technology may be applied to address all these constraints. The performance of different wireless communication systems is primarily improved by the employment of DL methods. Current communication systems heavily utilize DL-assisted NOMA technologies for a variety of uses. The authors in [19] examine how such systems are used in the literature to assess their contribution to system performance improvement and the various difficulties that arise in such systems when trying to change their design or create technologies optimized for 5G network performance. In recent studies, DL methods have been applied in the NOMA system to enhance the system’s functionality. Figure 2 shows a NOMA-VLC system with a DL-based signal demodulator to successfully attenuate nonlinear and linear distortions.

### 1.2. Scope and Contributions

Readers interested in reading DL-based NOMA should read this review. This paper offers an in-depth analysis of existing research works on DL-based NOMA. The purpose of this review is to assist readers with a thorough understanding of the DL methods in NOMA systems from a variety of perspectives. It provides a comprehensive discussion of key performance indicators for DL-based NOMA. It also explains the integration of emerging technologies, such as MEC, IRS, OFDM, MIMO, SWIPT etc., into DL-based NOMA. Finally, it explains a number of potential challenges and future research areas for further breakthrough novelties.

### 1.3. Organization of the Paper

This work is arranged as follows. Section 2 discusses the associated DL-based NOMA research contributions. We have discussed previously reported works and summarized some studies in a Tabular form. Section 3 covers the key aspects of the practical implementation of DL-based NOMA. We have briefly highlighted resource allocation, power allocation, channel state information, successive interference cancellation, user fairness, channel estimation, etc. Section 4 discusses the integration of DL-based NOMA with several emerging technologies, including mobile edge computing, OFDM, MIMO, intelligent reflecting surfaces and SWIPT. Section 5 highlights several research challenges encountered while using DL in NOMA. Section 6 concentrates on future research directions. We highlight several research aspects which require future research efforts. Lastly, Section 7 brings the paper to a close.

## 2. Related Work

Cell outage compensation in NOMA-aided networking has been disregarded despite all the efforts, and it has to be researched. In [5], the authors take into account the issue of a joint allocation scheme and user interaction. Traditional optimization-based numerical algorithms can be used to tackle the problem of power allocation in a NOMA-based system. In [20], power distribution in the uplink and downlink were modeled as convex optimization issues, with closed-form methods provided. In [21], an imperfect NOMA scheme with interference residue and receiver sensitivity from non-ideal decoding was investigated. An iterative approach was developed to address the resource allocation optimization issue, which considered a trade-off between proportional fairness and maximum throughput. These optimization-oriented strategies, however, must be repeated anytime network parameters alter over time. As a result, they suffer significant online computing costs and substantial overhead, impeding real-time development. To address the power allocation problem in NOMA systems, meta-heuristic techniques, including particle swarm optimization [22] and genetic algorithms [23], have been applied. However, they have the same limitations as the previously stated optimization-aided numerical techniques. Learning-based techniques have recently been developed in light of these constraints. RL is a prominent strategy for dealing with the problem of uncertain and variable environments [24]. RL-based strategies for power allocation in an outage compensation situation were put forth in [25,26]. However, one disadvantage of RL-based techniques is that they may take a very long time to converge as they attempt to identify the best answer through the interaction of their environment via trial and error. Because of this, they are less desirable in real-world cell outage compensation conditions when the network must respond quickly to a catastrophic loss and keep customers connected. On the other hand, unsupervised learning methods do not make use of the data that can be obtained when a network is operating offline. In [5], the authors suggest a supervised deep neural network (DNN) to compensate for the cell outage compensation issue by using the data that may be acquired offline and attain substantially reduced online computing complexity. For a variety of resource allocation issues in wireless networks, including beamforming [27], subchannel assignment [28], power control [29,30], and user association [31,32], it has been demonstrated that DNNs are a potential method for approximating the best policy. While DNN-aided resource allocation in wireless connections has been the subject of several studies, none have looked at how it may be used to address the issue of cell outage compensation, which is the subject of new research works.

The review papers [33,34,35] offer a thorough analysis of DL-aided interaction. The evolution and significance of DL are examined. The authors discuss how DL compares to potential wireless technologies. They also outline the potentials, difficulties, and future research directions for DL in a wireless environment. They show how the DL-aided NOMA system has improved spectrum efficiency, channel state estimation and system capacity. The discussion of massive MIMO’s direction of arrival (DoA) and channel estimation applies to DL-assisted MIMO systems. They provide an analysis of DL performance in mmWave communication under conditions of extremely high-power consumption and constrained link gains. [36] discusses NOMA’s function in the communication system. The full advantages of NOMA and NOMA in combination with several technologies, including mobile edge computing (MEC), visible light communication, energy harvesting, physical layer security (PLS), cognitive radio (CR), mmWave communication, cooperative communication, and massive MIMO are provided. Additionally, it exemplifies how studies on NOMA’s cohabitation with other systems are headed. In [37], a thorough study of downlink NOMA is given while taking into account networks with two to k users per cell using orthogonal multiple access (OMA) approaches. It goes through NOMA’s key characteristics, which raise the system’s spectrum efficiency. The discussion goes into great length on several aspects and difficulties in the NOMA system, such as SIC, inter-channel channel interference, channel state information (CSI), and power allocation factor. Additionally, the effectiveness of DL and machine learning (ML) for the next-generation NOMA system is examined. A review of the NOMA-DL potentials may be found in [38]. The study presents a thorough analysis of the difficulties in the wireless system and mentions the potential of DL. It is divided into sections on resource distribution, signal decoding, and the design of signal constellations. The scope and difficulties of DL-aided NOMA in wireless systems are discussed in that review paper. In-depth reviews of recent cellular and IoT network studies are provided in [39]. The authors talk about the difficulties with network coverage expansion, user clustering, interference control, and channel access for highly linked devices. In addition, the basics of DL approaches and the DL algorithm’s application to resource management for IoT-enabled devices are examined. The different types of DNNs and their most recent developments to the issues with wireless systems are thoroughly examined. The examination advances knowledge in devices-to-device communication, heterogeneous networks, NOMA, and MIMO, which are part of the next generation of this technology. Table 1 summarizes the recent reviews on DL-based NOMA systems.

**Table 1:** Summary of recent reviews on DL-based NOMA.

Reference	Focus Area	Research Contribution
[11]	A comprehensive review of DL-based NOMA	This study surveys DL-based NOMA techniques, benefits, and challenges of DL integration with emerging technologies. It briefly discusses future directions for DL-based NOMA systems.
[19]	DL-based NOMA for 5G networks	It discusses DL-based NOMA models exploited for user detection, signal detection, channel allotment, power allocation and resource allocation. It also highlights the shortcomings and benefits of DL approaches to solve NOMA challenges.
[38]	Performance analysis of DL-based NOMA	It outlines DL-based NOMA, its challenges, and potential benefits.
[37]	Overview of NOMA	It focuses on NOMA’s impact on multi-cell networks.
[36]	Advantages of NOMA	It comprehensively discusses the benefits of NOMA and the integration of cognitive networks, MEC and MIMO.
[40]	DL’s impact on wireless communication	It discusses DL-based sum rate maximization for NOMA
[41]	NOMA in next-generation multiple access	It discusses NOMA as a promising candidate for 6G networks. It also discusses associated research opportunities and future visions.
[42]	DL for downlinking MIMO-NOMA systems	This article surveys DL in SP blocks of downlink MIMO-NOMA systems. It briefly outlines possible future research directions.
[43]	DL-enhanced NOMA transceiver designs for massive MTC	It comprehensively discusses state-of-the-art challenges and future directions for DL-enhanced NOMA systems from the perspectives of online adaptability and reconfigurability toward the ever-changing environment in future mMTC.
[44]	DL-based power allocation in NOMA	It provides a review of the power allocation optimization problem through DL methods. It also outlines various future research directions.
Our Work	DL-based NOMA	In this study, we comprehensively discuss state-of-the-art, key performance indicators, challenges, and future directions for DL-enhanced NOMA systems. We also discuss the integration of emerging technologies such as MEC, MIMO, OFDM, SWIPT and IRS with DL-based NOMA systems.

## 3. Key Aspects for Practical Implementation of DL-Based NOMA

In this section, we have discussed several key performance indicators which play a major role in the practical implementation of NOMA systems.

### 3.1. Resource Allocation

In NOMA, one resource block (RB) is shared by multiple users, and the SIC receiver is used to decode the user information at the receiver end based on the user’s channel gains. Interference between the users can be avoided by choosing proper power allocation algorithms. Otherwise, resource allocation issues such as user pairing and power allocation (PA) will arise. In user pairing, the users with less power are allocated with more channel gain, and users with more power are allocated with less channel gain to make channel fairness to all the users at the transmitter end. At the receiver end, the SIC receiver is used to decode the same. In this method, if the number of users increases, then the decoding complexity also increases at the receiver end. This is one of the major problems in user pairing. Along with this, another problem, i.e., if the users with high and low gain are transformed to mid-gain, then mid-gain users may be paired or may not, which leads to reduced channel capacity.

To overcome the user pairing issues, optimization techniques, game theory, machine learning and deep learning algorithms are proposed in the literature. The authors proposed an optimization method while pairing two users [45,46,47]. To optimize the user pairing, the channel gain should not be less than the predefined threshold. A strong channel pairing algorithm can increase the system capacity and fairness in user pairing. In [46], the authors used a new pairing concept, i.e., the highest channel gain users are paired with the next highest gain users. Different Game theory algorithms for multiple user pairing and machine learning algorithms for user pairing have been proposed in recent research. In [26], the authors proposed an RL-enabled joint power allocation and user pairing scheme. Through Q-learning, they were able to successfully implement both power allocation and user pairing with reduced computational complexity. In [47], the authors introduced an optimal power allocation technique with a given sub-channel assignment through a closed-form approach. Considering this, a traditional deep reinforcement learning (DRL) algorithm named Deep Q-Network (DQN) algorithm is used to investigate the optimal user pairing scheme. The DQN algorithm provides better performance of the feature extraction ability and higher learning efficiency than conventional reinforcement learning (RL) schemes. 

### 3.2. Power Allocation

**Figure 3:**
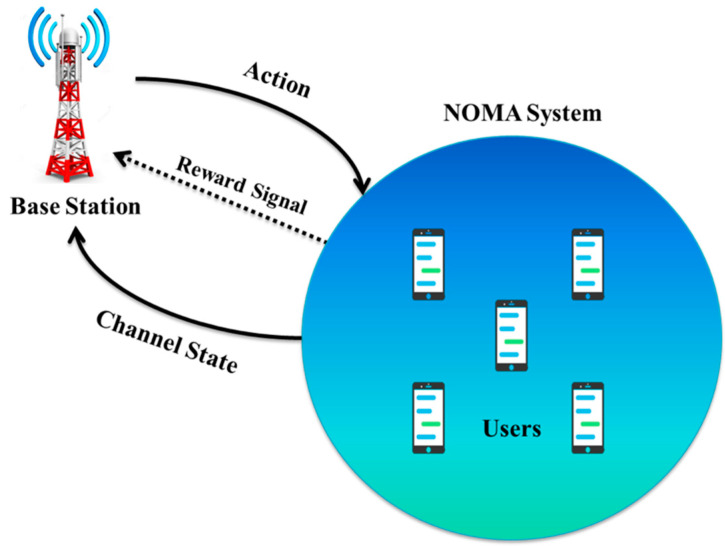
Power allocation and channel assignment of DL-based NOMA system.

One crucial challenge is how to allocate power when there are limited resources to make the most of the benefits of the NOMA system. It has been established that this issue of optimum power allocation is NP-hard, indicating that it is impractical and expensive to study all possible channel assignments to find an ideal solution. As a result, several methods have been put forth by researchers to deal with this issue. Solutions include distributing power for a downlink single input and single output (SISO) NOMA system [48], distributing power for the most equitable distribution of users [49], and distributing power for the most energy-efficient use of resources [50]. Deep learning techniques must be used because several solutions have been demonstrated to be less than ideal. A thorough literature assessment of deep learning-based approaches to the power allocation concern will be provided in sufficient depth in this section. Utilizing DL in NOMA, deep neural network generic architecture efforts are at the forefront of current technological advancements in power allocation. To distribute power to consumers in the best possible way, [35] suggests a deep reinforcement learning (DRL) method; specifically, an artificial neural network (ANN) is employed to perform channel assignment. The system model is based on BS and several users in a downlink NOMA scenario. Users serve as the performance environment for the deep learning algorithm, which treats BS as an agent. To allocate resources and channels to users, BS first chooses a task (channel assignment) from a set. A feedback signal is then provided towards the BS to help assign users in the following transmission based on the users’ responses. The three crucial parts of this process are the status space, action space, and reward function. The channel information is responsible for the state space. The agent (BS) chooses a single channel for data transmission for a single user in the action space. The collection of actions is constrained to meet the requirements of user channel allocation, so each user is associated with a unique action. After the user acts, the allocation procedure is complete. The signal returned to the BS as a result of a failed or successful transmission at the conclusion of each time slot is the reward function, as shown in Figure 3. The data rates each user experiences and is sent by the BS to make up the signal. The goal of [51] is to maximize this incentive signal and, in turn, optimize each user’s data rate. The acquired findings provide a sum-rate comparison of Joint Resource Allocation (JRA) without downlink versus JRA with DL, where the non-DL variant is significantly outperformed by the DL counterpart. [52] suggests a power allocation plan that uses DL approaches to optimize the system sum rate in a downlink NOMA environment with an incomplete SIC. The algorithm for finding the best power allocation is exhaustive. In a recent study [44], a power allocation approach for imperfect SIC to enhance the experienced system sum rate is suggested. The suggested technique extensively uses deep learning to predict the ideal power allocation criteria. We have summarized the research contributions on DL-based power allocation methods in NOMA in Table 2.

**Table 2:** Summary of studies on DL-based power allocation types in NOMA.

Reference	Technique	Model	System Description	Model Limitations	Research Contribution
[35]	Reinforcement	Deep neural networks	Physical layer framework, BS with multiple antennas and multiple single-antenna users	Complicated DNN, the data set acquisition and model selection problems	Introduces an efficient, cutting DL-assisted 5G and beyond communication.
[48]	Reinforcement	Artificial Neural Networks	Attention-based neural network exploiting an encoder-decoder structure,	The key problem is how to allocate limited resources to multiple users	Introduces a method to optimally allocate transmission resources through an attention-assisted neural network for channel assignment.
[50]	Unsupervised	K-mean	K-means-based online user clustering algorithm	It does not consider more complicated clustering algorithms that are robust to noises and outliers	Satisfy the total transmission power sum-rate maximization issue until the QoS demands of the users.
[52]	Reinforcement	Q-learning	A single base station in a NOMA system equipped with multiple antennas contending with a smart jammer	It considers a simplified jamming scenario. Theoretical analysis of more practical scenarios is still needed.	In the occupancy of a jamming device, the BS carries out our execution of power distribution. The process is developed as a game with a zero-sum outcome.
[53]	Reinforcement	Special Neural Network	Single BS using cooperative reinforcement learning algorithm for adaptive power allocation in D2D communication	It considers a single-cell setup, while a multi-cell setup is still required.	Considering the OFDM system without NOMA, maximize overall SU interference while keeping data rate and power.
[54]	Reinforcement	Q-learning	Dynamic multichannel access problem, where multiple correlated channels consider an unknown joint Markov model	Computationally expensive, DQN is not easy to tune, and more realistic and complicated scenarios such as multi-user systems are not considered.	Channel switching technique for realizing dynamic systems.
[55]	Supervised	CNN	The power control scheme in D2D communication in multi-channel based on CNN	Compared with DNN, it still lacks fitting ability.	Maximization of throughput by configuring the power.
[56]	Reinforcement	Two DNNs connected fully	DNN-based multi-channel cognitive radio networks, where the secondary user uses channels without interference from the primary user.	This system does not consider an optimal DNN structure.	Considering an underlay cognitive radio network, it focuses on maximizing spectral efficiency. Keeping the interference of primary users below the threshold, the secondary users provide high spectral efficiency.

### 3.3. Channel State Information

Practically speaking, channel state information (CSI) significantly influences the NOMA system’s performance, and several efforts have been made to implement channel estimation using NOMA scenarios [57]. In [58], a new linear estimator was developed to maximize the average effective signal-to-interference noise ratio (SINR) of the strong user, with a finite SINR required for the weak user to identify the CSI. Meanwhile, several researchers are looking at NOMA-based solutions in various CSI circumstances because the CSI is difficult to collect using conventional approaches. Two power allocation techniques and the performance of NOMA in an incomplete CSI environment were reported [59]. Furthermore, using uplink NOMA systems, the researchers demonstrated that insufficient CSI causes improper decoding and additional interference with the intended signal [60].

Consequently, how to efficiently collect flawless CSI is a crucial challenge in NOMA-aided approaches, and new techniques must be used to address this issue. Although numerous recent research contributions have developed various reliability and sum data rate optimization methods, these techniques demand high computational complexity because of the nonlinear optimization. They are unable to produce an associated power allocation mechanism against a given CSI. In particular, virtually all of the key benefits of NOMA techniques rely mainly on CSI; as a result, several strategies have been presented in previous studies to further enhance the effectiveness of channel estimates [61]. Conventional approaches, however, are unable to trace the alteration in the channel state in real time due to the complexity of the channel conditions in multiple-user systems [62]. Usually, the drastically fluctuating channel characteristics cause CSI acquisition to be disrupted and the NOMA system efficiency to suffer.

Nevertheless, nonlinear reconstruction techniques are inevitable since the channel sparsity trends have been frequently taken for granted in previous studies. Therefore, super-resolution direct arrival (DOA) estimates, and signal identification cannot be accomplished using standard approaches since they are inefficient and unreliable. The NOMA system has recently been enhanced with a promising machine learning (ML) approach to enable the auto-detection of the CSI. The DL [63] idea, introduced in 2006 and a typical branch of machine learning, is a particularly effective technique for managing large amounts of data and resolving challenging nonlinear issues. A few earlier papers [64,65,66,67] included DL in communication in relation to the physical layer, channel coding, and MIMO. The intriguing system that incorporates the DL into the OFDM context has been demonstrated in [68], and its outstanding performance in the context of signal recognition and channel estimation has been confirmed. DL has also been used in traffic monitoring systems, which work admirably [69,70,71]. Additionally, DL-based communication systems have shown certain benefits in terms of security, BER, and throughput performance. 

### 3.4. Successive Interference Cancellation

The drawbacks of SIC might also be addressed using the DL technique. Due to the SIC receiver’s poor cancellation, overall capacity decreases [42]. As a result of different hardware limitations, decoding and canceling may be faulty in real-world systems, making SIC possible. The performance gain of NOMA may be enhanced by using SIC at cell-edge users, as demonstrated by the authors in [72]. The creation of an easy-to-use, effective SIC receiver is essential to NOMA. Multi-stage SIC lowers multi-path fading and BER. The performance of the system is impacted by the signal’s decoding sequence. High signal-to-noise ratio (SNR) signals are initially deciphered. The performance of the SIC receiver is enhanced by a low complexity, highly effective power allocation algorithm [73]. A real implementation’s non-idealities and flaws cause error propagation in SIC, which is utilized to decode and identify desirable signals. Due to the signal processing required for SIC, receiver complexity increases as user equipment (UE) numbers rise. A deep neural network (DNN) is used in [74] to approximate the SIC receiver. In the MIMO-NOMA system, the combined optimization of precoding and SIC decoding minimizes the total mean square error between the user’s intended signal and their decoded signal. Users and their sub-bands are grouped according to the status of the channel, ascending. The binary dislocation principle pairs them (BDP). As a result, users with excellent and bad channel conditions will be paired.

A sub-band that satisfies user demands is selected. EP at the receiver can be removed if the signal-to-interference-plus-noise ratio (SINR) difference among a pair of users in the sub-band is sufficiently great. Users that share a band are given authority by BS. The minimum mean square error (MMSE)-SIC method with Interference Rejection Combining (IRC), which analyzes noise and interference independently and enhances average channel capacity and, therefore, system performance, can be used to attain the best performance at the receiver [75]. Some recent works are based on the theory that most EP-related problems in SIC may be handled by appropriately grouping or clustering users. Additionally, by concurrently optimizing precoding and SIC decoding using DNN and domain-specific information, the mean square error (MSE) value between the intended and decoded signals would be reduced to the absolute minimum. A comparison of reported studies on NOMA is presented in Table 3.

**Table 3:** Comparison of studies reported on SCI/CSI in NOMA [76].

Context	[77]	[78]	[79]	[80]	[81]	[76]
CR-NOMA	Yes	Yes	Yes	Yes	Yes	Yes
Downlink	Yes	Yes	Yes	Yes	Yes	Yes
Uplink	-	-	-	-	-	-
Imperfect SIC	Yes	Yes	Yes	-	-	Yes
Imperfect CSI	-	-	-	-	Yes	Yes
OP analysis	Yes	-	Yes	Yes	Yes	Yes
Throughput	-	Yes	-	Yes	-	Yes

“-” means this feature has not been considered in those references.

### 3.5. User Fairness

**Figure 4:**
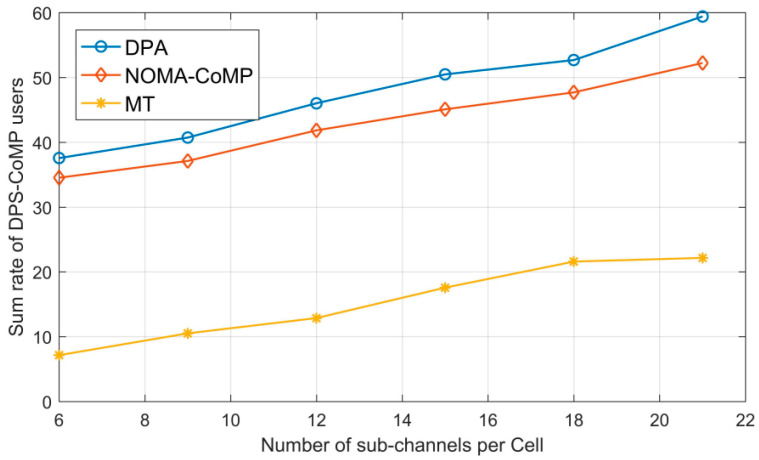
Sum rate of DPS-CoMP users (Mbps) versus the number of subchannels per cell [82].

Several recent studies [82,83] have discussed the benefits of employing NOMA. This increases system throughput, spectrum efficiency and user fairness. We can also obtain an extremely highly reliable connection. Time and frequency resources are distributed to users in the spatial domain through the power domain or code domain NOMA [79]. A recent work [82] uses a deep learning algorithm to ensure user fairness by dividing users into low-rate and high-rate requirement users considering their mobile phone usage habits. The authors consider a NOMA system with DL-based coordinated multi-point (CoMP), used in 5G cellular networks to guarantee the rate requirements from the different edge users. Figure 4 evaluates the performance of user sum rates in dynamic point selection CoMP (DPS-CoMP) subchannels and the number of cells subchannels. Figure 4 shows that the user sum rate in the DPS-CoMP subchannel of each cell in the DPA algorithm, the NOMA-CoMP algorithm, and the maximum throughput (MT) algorithm all increase as the number of subchannels increases. In the NOMA power domain, power distribution among users varies depending on the channel characteristics and user-specific channel quality. As a result, consumers located far from the BS will receive more power, and vice versa. According to the works cited in [84], fairness for NOMA in 5G is highlighted by the fact that, in downlink mm-wave NOMA, various data from all users is thus overlaid in the power domain at the transmitter, and the SIC is performed at the receiving side. By integrating SIC and superposition coding (SPC) at the transmitter end and receiver side, respectively, we may use NOMA to increase spectral efficiency. The max-min fairness of both the average CSI and instantaneous CSI is also discussed in this literature. Considering the interaction between NOMA and cooperative transmission, the integration of NOMA with several emerging 5G technologies, the correlation with other NOMA variants, and the resource control of NOMA, the authors in [85] focus on state of the art in power-domain multiplexing-based NOMA. Considering the hybrid beamforming system described in the article [85], which employs phase shifters and sets of switches, down-converter, LNA, ADC, and DAC are components of the radio frequency (RF) chain. The price of the system rises along with the number of RF chains. To decrease the number of RF chains, the hybrid beamforming approach is applied, and the system’s price will be reduced as a result. 5G deep learning systems have been researched in the literature. Power allocation, DoA estimation [86], physical layer security [87], channel estimation [88], energy optimization, etc., are all included in the program, which significantly addresses user fairness issues in NOMA.

### 3.6. Impulse Noise

#### 3.6.1. Impact of IN in NOMA

Numerous obstacles to the adoption of NOMA systems have been raised by the broader literature on NOMA, which primarily occurs with respect to next-generation networks like the Internet of Things (IoTs) and smart grids. Analysis has also been done on the impact of IN on the NOMA downlink [89,90] and uplink [91] systems. The NOMA uplink systems’ outage performance in the occupancy of IN is given in the research work [91]. Analytical findings and comprehensive Monte-Carlo simulations were used to verify the NOMA system’s sensitivity to IN. The effect of IN on the cumulative rate capacity of NOMA downlink systems was given by the authors in [90]. The real loss from IN was calculated using a particle IN scenario. The authors also examined how well NOMA performed across Rayleigh-fading channels with composite noise (impulse with AWGN). A union constraint on the bit error rate (BER) was developed using the pairwise loss of bits (LoB) formula. The research study quantified the variance in channel conditions that NOMA users encounter when there is composite noise. The operational reduction of NOMA-aided IoT networks caused by IN was examined, and a mitigation approach was suggested in [92]. For acquired OFDM symbols generated from the power domain multiple-NOMA (PDM-NOMA) strategy, a multistage nonlinear solution based on deep learning was presented.

#### 3.6.2. IN Mitigation Techniques

**Figure 5:**
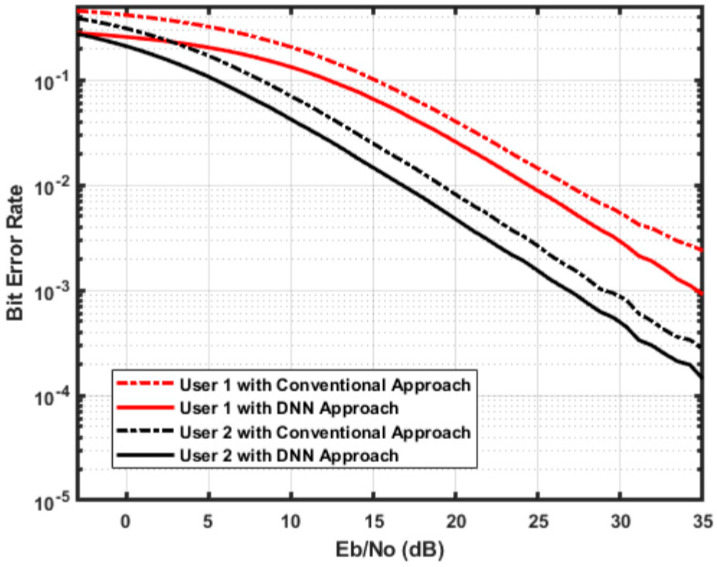
Performance of NOMA user pair through DNN-based technique [89].

The threshold-aided IN technique is defined as a memoryless nonlinear mitigation strategy that comprises blanking [93], clipping [94], and clipping/blanking [95]. In this method of mitigation, the high amplitude and short duration of IN are studied by employing a threshold whose adaptation seems difficult. The authors of [96] describe a threshold optimization strategy considering the Neyman-Pearson criteria. In [95], the authors presented a mathematical solution for IN mitigation utilizing clipping and blanking. In [97], a comparison of numerous analog domain processing strategies for IN mitigation demonstrates that threshold value selection is the most important aspect for enhancing the efficacy of threshold-assisted nonlinear techniques. Once the threshold varies due to channel circumstances, the model gets mismatched. As a result, extremely impulsive environments have a negative impact on the effectiveness of all conventional threshold-based approaches. In [89], the authors have successfully used DL approaches for IN mitigation. Figure 5 presents the DNN performance for IN mitigation in User 1 and compares it with User 2. User 1 uses SIC to reduce inter-user-interference. Thus, it will suffer from IN only. While User 2 is affected by both IN and inter-user interference. Therefore, it has variant BER performance according to SNR values. The results show that the DNN approach can be effectively utilized to overcome IN.

### 3.7. Transceiver Design

A recent study [43] focuses on explaining how deep learning aids in overcoming the NOMA as mentioned above difficulties. The multiuser receiver (Rx) is initially improved using deep learning from a model-driven and data-driven perspective. The authors briefly explain how deep learning may enable the optimization of end-to-end NOMA transceivers with practical transmitter (Tx) restrictions or domain knowledge. End-to-end learning is used in NOMA to integrate computation and communication. The authors investigate how deep learning can extract and use upper-layer data for transceiver design. They conclude by outlining some exciting new avenues for deep-learning-enhanced NOMA in mMTC.

#### Multiuser Detection Design [98,99,100,101,102,103,104,105,106]

**Figure 6:**
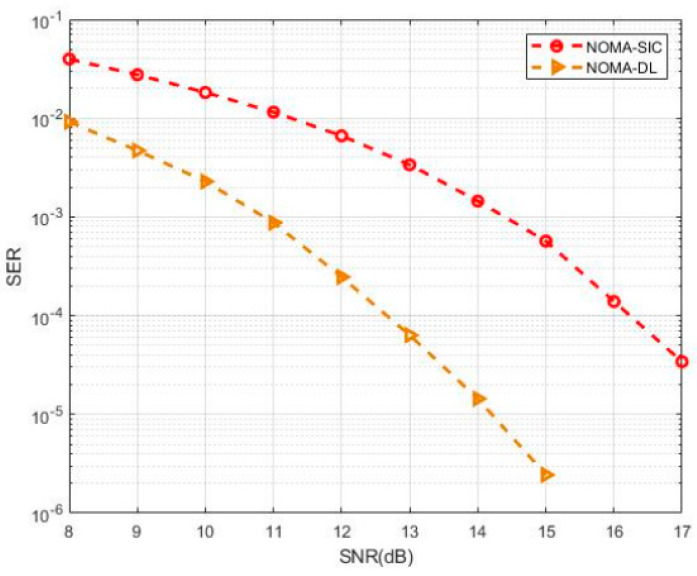
Performance comparison of MIMO-NOMA-DL and MIMO-NOMA-SIC [103].

Different users’ signals are sent in a non-orthogonal fashion in NOMA. Generally, multiuser detectors (MUDs) are used at the receiving end to differentiate between the overlapping signal streams, thereby minimising inter-user interference (IUI). For several NOMA systems, state-of-the-art MUDs have been created, including parallel interference cancellation (PIC), sequential interference cancellation (SIC), and message-passing algorithms (MPA). Unfortunately, multi-user detection still lacks a unified signal processing framework. By using DNN to improve MUD, we may get a more unified architecture, higher detection accuracy, and shorter processing times. DNN-based concepts may be roughly divided into two distinct camps: data-driven and model-driven. Vanilla DNNs are used in a data-driven strategy, which reduces the time spent on design but increases the amount of data needed for training. Alternatively, a model-driven method uses domain-specific knowledge from NOMA to reduce the need for data and increase learning efficiency. In a recent study [103], the authors propose a DL method that automatically analyzes the CSI of the communication system and detects the original transmit sequences. Figure 6 shows the symbol error rate (SER) and SNR curve of the numerical simulation. The proposed MIMO-NOMA-DL reached 12.6 dB, whereas the traditional scheme reached 16.2 dB—a difference of approximately 3.6 dB. The authors used powerful DL tools to perform accurate signal detection rather than traditional complex signal processing for channel estimation and demodulation.

### 3.8. DL for Channel Estimation

In a MIMO-NOMA system, an accurate channel estimate is crucial since it influences the system’s performance. Appropriate CSI is necessary for interference cancellation at the receiving end. DNN may be a good option for calculating precise CSI and channel estimates. The researchers of [103] created an algorithm that automatically assesses and seeks the best logical plan for AWGN channels and MIMO Rayleigh fading channel-state information to recover the signal. For every conceivable scenario, including power allocation parameters, it was demonstrated that DL-based approaches might outperform SIC receivers in terms of symbol error rate (SER) performance. Channel estimation and detection were carried out in batches throughout the training phase. In the testing phase, channel error was included, and the authors investigated how the DL method behaved when the estimated CSI and the actual channel state were different. Throughput is decreased because channel estimate errors cause residual and SIC decoding errors. Impact channel estimate error and reference signaling are reduced by the transmission rate back-off method (in which the transmission rate is regulated). Random beamforming is a useful technique for lowering CSI feedback [107]. Since flawless interference cancellation depends on the correct CSI estimate, NOMA system performance is impacted. Practically speaking, it is challenging because of the complicated fluctuations in channel conditions brought on by high mobility. Utilizing the spatial diversity of massive MIMO, DL methods may be utilized to evaluate the DOA and real-time channel estimates. The sparse features may be fully extracted and efficiently used in the DL technique to learn the entire system. DL also performs better than traditional approaches when SNR is high [35].

The articles make it clear that estimate accuracy is a performance parameter and that DL is highly preferred to estimate CSI in real-time with less complexity and pilot overhead than the conventional alternatives. Large datasets with different channel conditions are still difficult for supervised learning, and offline network training takes time. Its efficacy in cases with high mobility is constrained since it is challenging to predict the channel.

### 3.9. DL for Beamforming and Selection

The performance of 5G technology is also determined by the beamforming process. A quick unsupervised learning-based beamforming design methodology has been put out by authors in [108]. In this approach, DNN is trained offline and provides real-time assistance for simple neural network tasks once it is online. DNN in the downlink records the channel’s characteristics, takes the channel coefficients as input, and produces a beamformer. Pruned DNN is used because it decreases the parameters and, as a result, the computational complexity and time required by DNN. The simulations showed that, although degrading with increasing SNR and transmit antenna count, deep neural network performance is comparable to that of WMMSE. In [109], DL-aided hybrid beamforming (HB) is suggested, where supervised learning and an autoencoder build the HB. Compared to other traditional beamforming methods, this approach performed better in the context of bit error rate. A recent study [110] introduces a novel MIMO-NOMA system that addresses partial CSI feedback. Channel quality information (CQI), the best beam, and beam correlation are used to cluster users. The user pair chosen for clustering had the greatest CQI differential and the highest beam correlation. HB is created through clustering. As an analog beamforming vector, the best beam from a high data rate user is selected. Thus, inter-cluster interference is decreased. For weak users, digital beamforming is used to reduce intra-cluster interference. Furthermore, the authors developed a system with efficient power allocation by optimizing the power differential between UEs in a cluster while subject to rate constraints. The system that was presented had a greater sum rate. With DL, choosing a beam is simpler. Using two optimal beam indices as inputs and an estimated power delay profile (PDP) as a label, the DL model is trained using supervised learning. Adam is used for optimization, cross-entropy is utilized as the cost function, and softmax activation is employed at the output layer, where the number of beams equals the number of neurons [111]. DNN may be used to perform beam selection and hybrid beamforming with little latency. Additionally, it produces better results from the perspective of summation rate and BER.

### 3.10. DL for Modulation and Signal Processing

**Figure 7:**
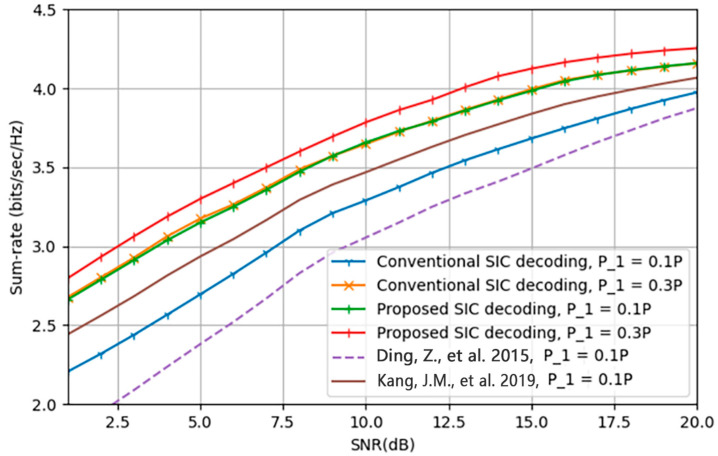
Sum rate versus SNR for the conventional and proposed SIC schemes with varying power allocations [98,119,120].

At high SNR, long-short-term memory (LSTM) and the deep residual network (ResNet) may achieve high classification accuracy. Still, the convolutional long-short-term deep neural network (CLDNN) and ResNet performed well at low SNR. Furthermore, principal component analysis and subsampling were used to minimize training time [112]. In the presence of faulty CSI, CNN for feature extraction and DNN for joint channel equalization and decoding have high accuracy. In terms of BER and decoding rate, DNN outperforms CNN [113]. A system that combines CNN and LSTM is thought to perform well in automated modulation classification (AMC) at varied SNRs [107]. For signal demodulation using Rayleigh and AWGN channels, CNN and a bidirectional gated recurrent unit layer known as a mixed neural network model are utilized. CNN is utilized to extract features, whereas RNN is used for time-series analysis [114]. In [105], the authors propose a deep residual network-based blind modulation detection technique that uses a noisy joint constellation as input. Wavelet denoising is used to increase constellation quality. To demodulate the signal, the SIC receiver at the distant UE requires information on the modulation mode. This technique significantly reduces signaling overhead while improving service quality in NOMA systems. However, for higher-order modulation, the constellation becomes more difficult. A CNN-based AMC with an extended symbol rate sequence and an estimated SNR is a near approximation to a maximum likelihood-based AMC (ML-AMC), learning from raw data and processing in parallel, making it quicker and better than feature-based approaches and ML-AMC [115]. A survey on DL in signal recognition reported in [116] highlighted the difficulty in developing an accurate and effective DL signal recognition system in coexistence. For modulation recognition under various channel impairments and datasets, a modified deep residual network (RN) has been deployed. This outperforms CNN in terms of efficiency. Transfer learning is employed to accelerate the suggested model. In addition, the authors compare the baseline approach and strongly boosted gradient tree classification for radio signal classification utilizing over-the-air observations [117]. In [118], the authors propose employing a single DNN for joint optimum MIMO signal detection and channel decoding. The suggested DNN model has the limitation of requiring training for different channel matrices as well as having a high decoding latency. A DNN that can handle multiple channel matrices with a single training is offered as a research path. For modulation categorization, signal identification, and decoding, prominent models include CNN, RN, LSTM, and customized DNN. In a recent work [119], the authors investigated a deep learning-based SIC scheme for NOMA communication systems and compared its performance with [19] and [120] as shown in Figure 7. The authors propose a convolutional neural network (CNN)-based SIC scheme to enhance the single BS and multiuser NOMA scheme. The proposed CNN-based SIC scheme can effectively mitigate losses resulting from imperfections of the SIC. The findings also indicate that the CNN-based SIC method can achieve good detection performance and relieve conventional SIC impairments.

## 4. Integration of DL-Based NOMA with Emerging Technologies

### 4.1. Mobile Edge Computing (MEC)

Tasks may be completed at the edge server due to mobile edge computing (MEC), which is seen as a potential method of offering low-latency computing services. However, because MEC servers often have limited compute resources due to the high deployment costs, collaborative edge computing, which may make use of dispersed MEC servers, provides an effective way to increase system processing capacity [121]. Therefore, it is important to look into the computation offloading problem in collaborative edge computing, which includes the task offloading problem, which determines which server is chosen to complete the task, and the resource scheduling problem, which establishes how many communication and computation resources are allotted to the task. Numerous studies have recently examined the compute offloading issue in NOMA-MEC systems [122,123]. The majority of current approaches concentrate on the short-term scenario when the task request is predictable, and the environment of the system remains stable [124,125,126,127,128]. When using this strategy, task offloading, and resource scheduling decisions must be promptly updated whenever the system environment alters. This strategy typically involves significant computational complexity and results in severe performance degradation because it fails to recognize the relationship between various scheduling time slots in a dynamic IIoT environment. To better understand dynamic computation offloading schemes, see [129,130,131,132]. These plans, however, do not take into account the collaboration between several edge nodes, which might lead to load-balancing issues. The work offloading strategy for NOMA-based collaborative edge computing, as investigated in [133,134], is established based on users’ serving distances [135] or signal power [133], which does not inherently address the load balancing issue. Some works suggest a dynamic compute offloading method to fulfil the long-term quality of service (QoS) of delay-sensitive operations to overcome this issue and fully use the advantages of combining NOMA with collaborative edge computing in the IIoT.

**Figure 8:**
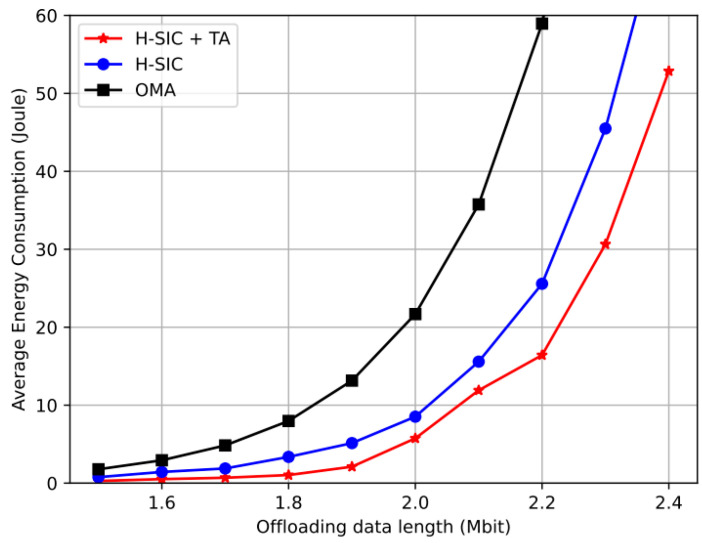
Average energy consumption versus the offloading data length [132].

**Figure 9:**
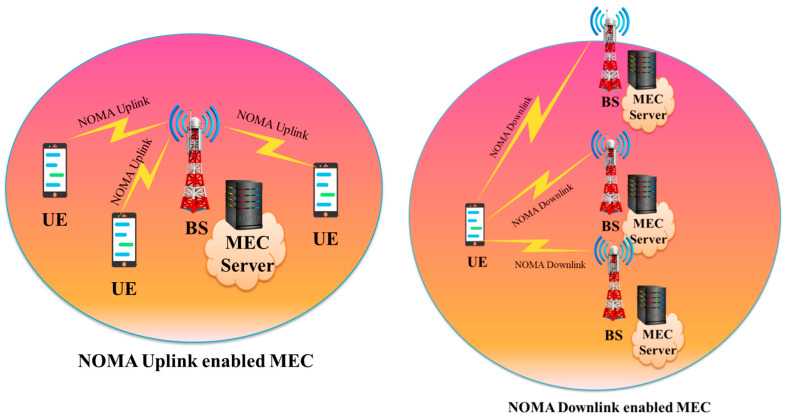
NOMA-MEC scenario for uplink and downlink.

In [136], the authors provide a DRL-based solution for supporting the binary computing offloading policy, which optimizes the task offloading choice and the sub-channel assignment to maximize task fulfilment. Every agent for an IIoT device uses a recurrent neural network (RNN) to forecast the load statuses of sub-channels and MEC servers; this information is then utilized to inform a Reinforcement Learning (RL) agent’s decision. The suggested prediction-based-DRL (P-DRL) technique is shown to be able to attain a greater task completion ratio than existing systems through simulation. In Table 4, we have summarized studies on the MEC scenario. In a recent work [132], the authors propose a hybrid SIC (H-SIC) scheme, which dynamically adapts the SIC decoding order among all NOMA groups. A DRL-based algorithm was proposed to obtain a close-to-optimal user grouping policy. The authors optimally minimized the offloading energy consumption by obtaining the closed-form solution to the resource allocation problem. Simulation results presented in Figure 8 show that the proposed H-SIC and H-SIC with task assignment outperformed the existing orthogonal multiple access (OMA) schemes. While Figure 9 presents the NOMA-MEC scenario for both uplink and downlink.

**Table 4:** Summary of studies on MEC scenario [137].

Reference	NOMA Incorporation	Number of Users	Offloading Mode	DL-Based Algorithm	Key Potential
[134]	Yes	Single user	Partial	Yes	It gives better performance as compared to FDMA-based MA-MEC
[137]	Yes	Multi-user	Partial	Yes	It speeds up convergence for better solutions than conventional optimization approaches
[138]	Yes	Single user	Partial	Yes	The DRL-based algorithm can achieve the near-optimal offloading solution fastly after enough learning
[139]	Yes	Multi-user	Binary	No	It increases the number of users to offload tasks and reduces the users’ average offloading delay
[140]	Yes	Multi-user	Binary-Partial	No	It outperforms the binary computation offloading mode, and NOMA outperforms TDMA in the context of computation efficiency
[141]	No	Multi-user	Binary	Yes	It can achieve near-optimal performance while substantially reducing the computation time by more than an order of magnitude than existing optimization techniques

### 4.2. OFDM-Based NOMA

Deep learning applications in error correction codes and signal processing [142] tend to involve polar code decoding [143], an auto-encoder in the physical layer [144], an auto-encoder for sparse code multiple access (SCMA) coding, and decoding [145], channel estimation and signal detection in OFDM [68], and an auto-encoder for data detection in NOMA [57]. After training, the deep learning-based technique has a computation time between 5 and 11 times faster than non-deep learning alternatives [145]. A recent study [142] compares radio resource allocation (time, subcarrier, power resource, etc.) of previous research in this field utilizing deep learning. Unsupervised learning, for example, using an auto-encoder in the physical layer [67], SCMA [145], and data detection in NOMA [57], in which the input and output of the network are the same, is not suitable for radio allocating resources. Previous radio resource allocation (subcarrier/time slot assignment) research has all focused on single-label classification issues, in which only one subcarrier (channel) may be allocated (labeled) to a single user. However, using NOMA, one subcarrier (channel) can be assigned (labeled) to two users. Standard DNNs are insufficient for multi-label classification. To circumvent this, assume all class combos to be different classes. This results in a combinatorial increase in the number of classes. Overtraining occurs when multiple parameters are trained on a limited training dataset [146]. As a result, new approaches for specific applications must be presented. Smart meter research [146], the multi-speaker direction of arrival estimate [147] and offloading for mobile edge computing [148] are all related publications. Researchers do not, though, apply deep-learning-based multi-label classification in NOMA systems for radio resource allocation.

### 4.3. IRS-Assisted NOMA

Due to their superior spectrum-, energy-, and cost-efficiency [149,150], intelligent reflecting surfaces (IRS) have been identified as a potentially game-changing technology for sixth-generation (6G) wireless communications [151]. Essentially, IRS is a cheap antenna array with a high number of tunable reflecting pieces [152]. IRS has the capacity to regulate the wireless communication propagation environment. In contrast, a number of tried-and-true methods, such as massive multiple-input multiple-output (massive-MIMO) and cooperative communications, solely focus on how the transceiver can adapt to the channel environment [153]. When the line-of-sight (LOS) between the BS and the users is obstructed by obstacles like buildings or mountains, IRS can be used to forge other pathways to maintain reliable connections and quality of service (QoS). In [152], the authors integrate NOMA with the IRS because they like its benefits, namely its high spectrum efficiency, and because research shows that it performs better than standard orthogonal multiple access [154]. Unmanned aerial vehicles (UAV), in contrast, are a promising 6G approach that can be employed to increase IRS adaptability. Previous research [155] optimized beamforming, phase shift, and UAV location for a 2-user IRS-UAVNOMA downlink system and found the best-closed form for beamforming.

Nevertheless, the time-varying multiuser situation is more like wireless communication networks. Therefore, it is challenging to tackle these non-convex joint optimization problems using traditional optimization techniques like convex optimization. In contrast, artificial intelligence (AI) techniques like deep learning (DL) and deep reinforcement learning (DRL) have been effectively used to solve a number of wireless communication challenges [156]. Wireless communication systems can learn by interacting with their surroundings using DRL-based methodologies instead of deep learning (DL), which requires many training labels. DRL is better because it is hard to get training labels in real-time wireless communication systems. Also, deep deterministic policy gradient (DDPG) is better than other DRL techniques like deep Q networks (DQN) for situations with a high-dimensional continuous action space.

**Figure 10:**
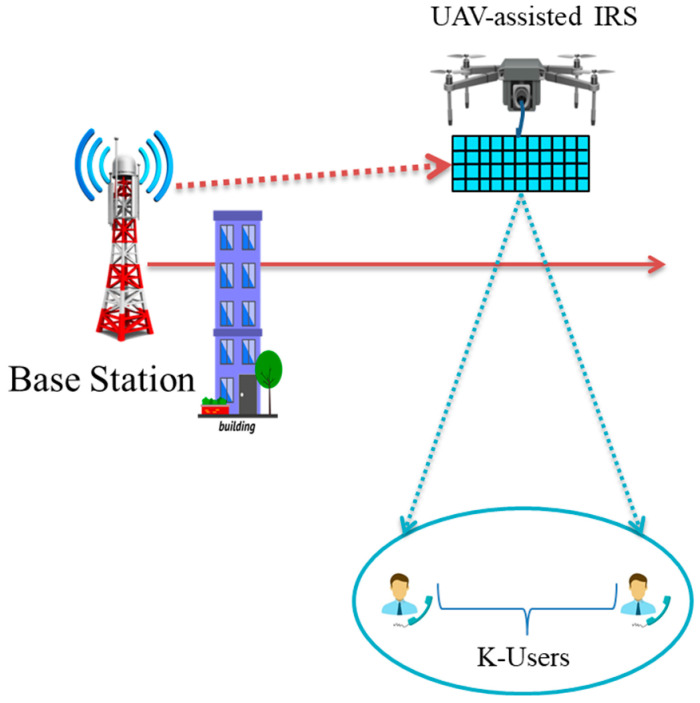
UAV-based IRS-assisted NOMA system.

In an article [151], the authors look at how DRL-based approaches may be used for the IRS-UAV-NOMA downlink system for multiple users, as shown in Figure 10. To maximize the efficiency of the BS’s power distribution, the IRS’s phase shifts, and the UAV’s horizontal position all at once, the DDPG algorithm is included in the DRL framework. The improved performance of the suggested algorithm is shown by computer simulations, which show both how fast it is and how well it can handle different inputs. A comparison of studies reported on IRS-assisted NOMA is presented in Table 5.

**Table 5:** Comparison of studies on IRS-assisted NOMA [157].

Context	[158]	[159]	[160]	[161]	[162]	[163]	[164]	[165]
NOMA system	Yes	Yes	Yes	Yes	Yes	Yes	Yes	Yes
OMA comparison	Yes	Yes	Yes	Yes	Yes	-	-	-
Achievable rate optimization	Yes	-	Yes	-	-	-	Yes	Yes
Op analysis	-	Yes	-	Yes	Yes	Yes	Yes	-
Hardware impairment	-	-	-	-	Yes	Yes		-
NLoS consideration	-	-	-	-	Yes	Yes	Yes	-
LoS consideration	Yes	Yes	Yes	Yes	-	-	-	Yes

### 4.4. MIMO-Assisted NOMA

Massive multiple-input multiple-output (MIMO) has been extensively researched as a promising method for enhancing the capacity of a communication network by merely adding more antennas [166,167]. The use of MIMO gives extra degrees of freedom for further performance optimization. Hence the use of MIMO over NOMA is quite interesting [168]. MIMO-NOMA is a strong option to help communication networks achieve even greater spectral efficiency and lower latency. MIMO-NOMA has generated a lot of attention because of its promising performance, and several MIMO-NOMA-based methods have been put forth. NOMA is included in beamspace MIMO in mmWave transmission, a novel approach with a high spectrum and energy efficiency presented in [169] to increase bandwidth and energy efficiency. Authors [170] explored a multi-cell MIMO-NOMA system in the downlink. They deduced a few interference alignments (IA)-based coordinated beamforming strategies where the beamforming vectors were mutually optimized at two BSs to reduce inter-cell interference and boost the throughputs of the cell-edge users. Furthermore, in [171], the authors investigate the energy efficiency (EE) problem in a millimeter wave massive MIMO system with non-orthogonal multiple access (NOMA). They formulate a power allocation problem aiming to maximize the EE under users’ quality of service requirements and per-cluster power constraint. Additionally, a unique MIMO-NOMA transmission approach was developed that outperformed both OMA transmission and the signal alignment NOMA scheme in terms of total power consumption [172]. This was accomplished by creating precoders and equalizers for users within the same group. Fundamentally, MIMO-NOMA systems have been shown to offer a better capacity and data rate than 4th generation (4G) MIMO-OMA systems. Power allocation algorithms have been studied in a number of ground-breaking studies. The authors in [173] investigated the cumulative data rates for the current power allocation strategies for a downlink MIMO-NOMA system with two users grouped into each cluster. For an indefinite rate pair obtained by MIMO-OMA, it was stated in [173] that a power splitting method was necessary to accomplish the rate pair by MIMO-NOMA with less power consumption. A multilevel security model-based opportunistic NOMA method for 3D MIMO channels was suggested. A range of security levels is assigned to the BS and users to increase the network throughput and secrecy capacity of MIMO-NOMA [174]. Based on the MIMO-NOMA system, the researchers in [175] suggested dynamically grouping the receive antennas at the users into a certain number of clusters and deriving power allocation solutions to increase the overall cell capacity. The number of clusters seems to be at least equal to the number of transmit antennas at the BS. The precision of the channel state information (CSI), which is required by SIC for each user, has a significant impact on the possible gain of MIMO. The existing power distribution plans are predicated on flawless CSI. Furthermore, it is very challenging to obtain the precise CSI in MIMO-NOMA systems owing to their unreasonably large complexity. The effectiveness of the present power allocation techniques suffers as a result. Despite sparsity information being essential for MIMO channel estimation and precoding, there have been few attempts to investigate the spatial degrees of freedom MIMO-NOMA provides. Most power allocation problems for MIMONOMA’s sum data rate optimization are NP-hard. The performance of the current power allocation solutions is constrained since the previously presented approaches can only derive suboptimal solutions.

**Figure 11:**
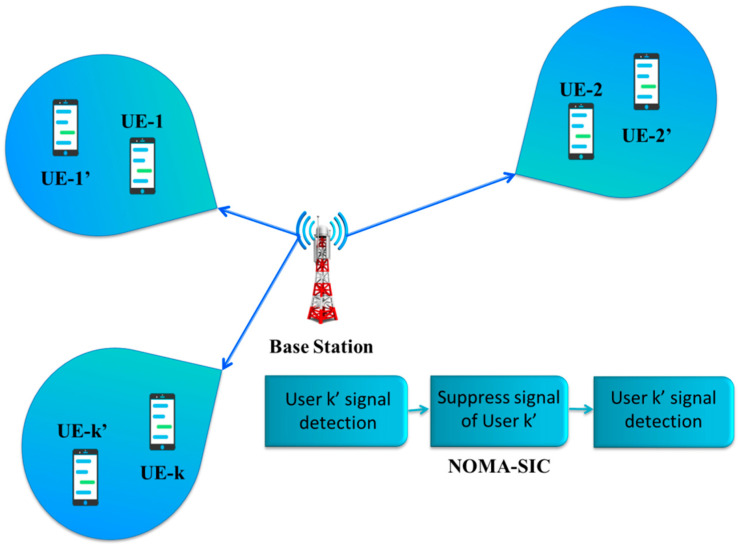
A MIMO-NOMA system with multiple clusters [166].

In light of these issues, it’s important to look at alternative theories that might improve the efficiency with which MIMO-NOMA systems allocate power. The deep learning approach, a subfield of machine learning, has recently been shown to be an extremely useful tool for handling large datasets and solving nonlinear issues. Although deep learning-based wireless communication is still in its infancy, its better performance has been proven by a number of ground-breaking experiments [50,57]. Initially introduced in [57], deep learning is now an integral part of NOMA, with impressive results shown for encoding, decoding, and channel recognition using a system built on deep learning. Also, deep learning has been implemented to improve massive MIMO channel estimates [86] and orthogonal frequency division multiplexing (OFDM) channel estimations [68]. Therefore, some recent studies present a deep learning-based MIMO-NOMA framework for optimizing the combined data rate and power consumption. In particular, numerous convolutional layers and multiple hidden layers are incorporated into the architecture for a powerful communication deep neural network (CDNN) [166]. The CDNN framework solves the issue of power allocation in MIMO-NOMA, allowing for a faster data rate and greater efficiency in terms of energy consumption by employing training algorithms that take advantage of deep learning’s powerful representation capabilities. Extensive simulations further demonstrate that the proposed CDNN framework achieves a higher total data rate and greater energy efficiency than conventional techniques, further supporting the claim that it is a good option to improve the performance of MIMO-NOMA in terms of power allocation. An illustration of the MIMO-NOMA system with multiple clusters is provided in Figure 11.

### 4.5. NOMA-Assisted SWIPT

**Figure 12:**
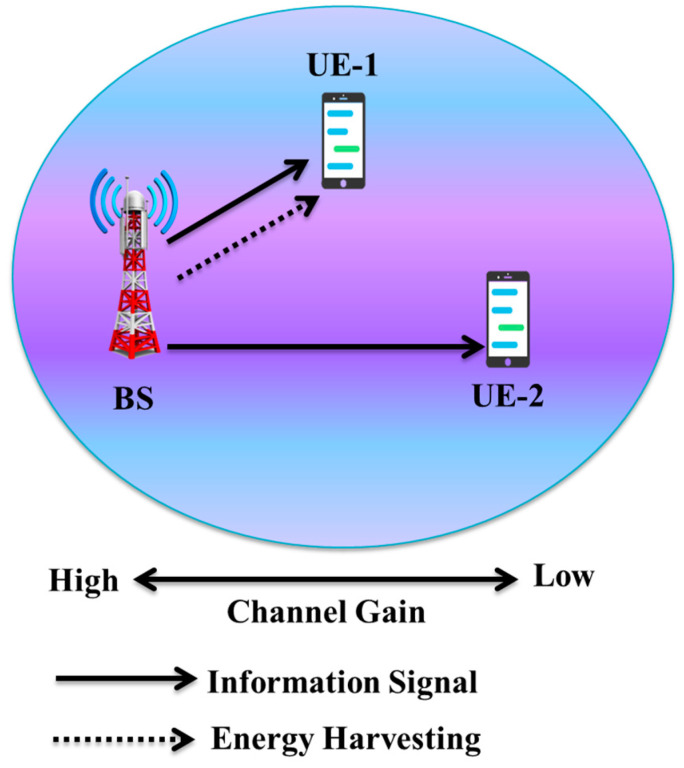
NOMA-enabled SWIPT.

By simultaneously gathering energy and information, SWIPT [176] aims to eliminate energy waste and increase the battery life of communication terminals. However, the current research on the SWIPT system tends to optimize them individually since the transmission rate and the gathered energy are incompatible [177]. As a result, it is important to investigate how these two goals trade-off. A lot of studies have been conducted on the use of SWIPT technology in various schemes, including heterogeneous cellular networks [178], two-way cooperative networks [179], multiple-input single-output (MISO) systems [180], multiple-input multiple-output (MIMO) systems [181], and OFDMA systems. SWIPT is also seen as a potential energy-efficient solution for 5G and beyond wireless networks. Numerous studies have investigated how to minimize terminal power consumption by combining SWIPT technology with the NOMA network. For instance, investigations on SC-NOMA systems based on SWIPT have been examined in [182,183]. In particular, the work in [182] explored the SWIPT in cooperative MISO SC-NOMA systems. It proposed a method of simultaneously optimizing the PS ratio and the beamforming vectors to optimize the data rate of the “strong user” while fulfilling the QoS criteria of the “weaker user.” Sum secrecy rate (SSR) maximization was examined in [184] in a SWIPT-based SC-NOMA system, and the numerical findings showed that performance improvements in terms of SSR over both traditional OMA and SWIPT-based OMA systems could be made. The NOMA-based SWIPT system is presented in Figure 12.

## 5. Potential Challenges

**Figure 13:**
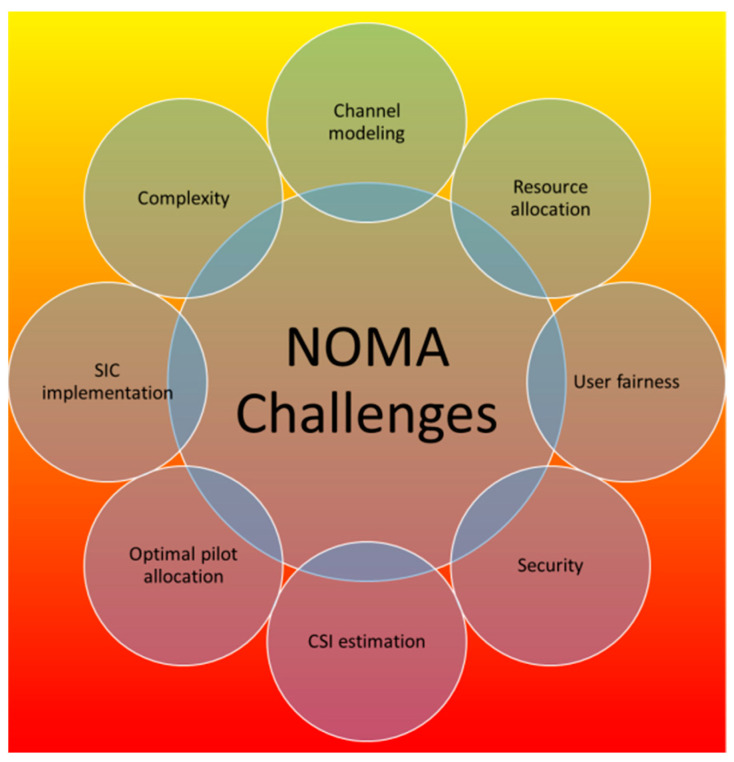
Basic challenges in NOMA.

NOMA systems are prone to several challenges, as presented in Figure 13. This section outlines the numerous difficulties encountered while using DL in NOMA. In addition, we offer the DL-aided NOMA system’s research guidelines.

### 5.1. Challenges in Resource Allocation

Creating a system with optimal resource allocation is difficult in a real-time scenario. The DL models should be trained using data relevant to users in a dynamic context. It is important to consider the computing expense to train neural networks in relation to network convergence, neurons, learning rate, and the number of hidden layers. The additional difficulties in the DL models may include fitting problems, e.g., underfitting and overfitting.

### 5.2. Challenges in Automatic Signal Detection

In a NOMA-based system, the detection of multi-users and decoding of the signal at the receiving end is a crucial procedure. A trained neural network can replace the SIC receiver to substantially enhance the performance of the communication system. Differentiating between distinct modulation symbols needs the development of a DL model. The application of reinforcement learning at the receiving end, which should pose a correlation of the detection with the time-varying channel condition, may result in automatic user detection.

### 5.3. Development of End-to-End Framework

DL might be implemented over the entire end-to-end system. When creating an end-to-end DL-assisted architecture, a large amount of labeled data is required to train the network, and each model must be connected to the next while taking channel impairments into account [185]. The recommended DL algorithms might alter based on the type of application. The algorithms can be monitored, unsupervised, or based on reinforcement learning, and power utility requirements depending on training should be considered.

### 5.4. Implementation of More Than Three-User Pairing in Real-Time

Significant attention has been paid to NOMA’s intriguing ability to serve many users inside a single resource block. However, it is clear from several real-world studies and applications that the three- or more-user pairing approaches and the two-user pairing idea are only taken into account in a small number of studies [19]. The fundamental limitation on the number of users multiplexing in a NOMA user pair is most likely caused by the need to retain user channel gains at the BS to carry out SIC at receivers. Furthermore, due to the increased need for interconnected devices, particularly for the Internet of Things, machine-to-machine, and massive machine-kind interactions, these vastly diverse networks must be maintained by developing esoteric multiple-user pairing techniques and methodologies. Therefore, it is necessary to create approaches that may cluster numerous users inside a given resource block to fully and effectively profit from NOMA, improve mass coverage, and fulfill future network requirements.

### 5.5. Uplink NOMA

Uplink transmission using 5G and its integration with new and current mobile technologies receive less attention than downlink transmission. As a consequence, the efficiency of our communication networks has decreased. Thus, it is important to investigate and recommend high-quality uplink transmission settings in NOMA from the perspectives of practical realization, security, and signaling.

### 5.6. Hybrid NOMA

5G and next-generation mobile networks are envisioned to run on a mix of MA techniques instead of the absolute replacement of the present OMA approach by NOMA. System performance and capacity advantages from NOMA are maximized only when paired users have dissimilar channel gains. Nevertheless, in cases where users had comparable channel gains, the literature’s pairing strategies resulted in a net drop in throughput. Instead of abandoning these users, it is preferable to employ or create more sophisticated DL technologies to help improve their performance.

### 5.7. Mobility

Users’ mobility in NOMA networks becomes a crucial issue that has to be addressed in additional studies, particularly for vehicular systems. Numerous studies primarily consider static surroundings, rendering these models unsuitable for use in wireless or mobile situations. Mobility impacts the maximum user pairing because it results in constant changes in users’ channel gains. Due to mobility, the semi-static NOMA channel requires to be further reprocessed to determine the maximal decoding order. Mobility also impacts user pairing in terms of the variety and quantity of components accessing a resource block. To make wireless NOMA work with the static frameworks that are currently available, new techniques are needed.

### 5.8. Multicell NOMA Networks

While many studies have been conducted on NOMA, the vast majority of attention has been paid to single-cell NOMA connections. Accordingly, further research is required in this area for real-world applications. While there are many advantages to multicell settings, interference concerns can reduce performance for users on the cell’s periphery. Microcell collaboration in multicell NOMA-provided heterogeneous networks will likely resolve this ancillary issue in addition to persistent intracluster and intercluster interference constraints.

### 5.9. Model Selection

When considering NOMA focusing on DL, the NN architecture stands out as a significant barrier. Several technologies and methods reliant on DL have been created using standard templates. While these models are geared toward DL-based NOMA applications, it is not certain that they will be successful. Generic models are expected to make it possible to implement such systems in practice. For real-world applications, generic models improve the ease of optimizing communication networks and save time and resources spent on selecting a suitable model. As a result, there is a pressing need for extensive research into creating such generic models to achieve overall performance improvement.

### 5.10. Performance Analysis and Learning Mechanism

The efficacy of DL-guided communication systems has been demonstrated for many different applications, including massive MIMO, channel estimation, coding, and decoding, among others. However, no rigorous mathematical proofs or dependable theorems are presented to verify the system’s accuracy. A firm theoretical foundation is crucial for comprehending the communication model and providing a foundation for network adaptation and the utilization of highly successful communication frameworks; therefore, this is where the bulk of our attention must be placed. Although it is common practice to transform the original input signals into different information representations to improve performance in the DL domain, achieving state-of-the-art performance in DL-based NOMA systems while changing the information representation styles is not guaranteed. While DL-based communication models are being developed, their optimal results and learning algorithms remain unclear. In addition, additional study is required before offering answers concerning the selection of training instances based on these networks.

### 5.11. Imperfect Successive Interference Cancellation

To separate user data or decode overlaid data for multiuser identification, SIC operations are often carried out on the receiver side of NOMA. No-interference multiple-access (NOMA) systems can greatly benefit from this procedure since it helps eliminate intra-beam interference. However, throughout the SIC method, information from users with high channel gains is taken on behalf of users with low channel gains. Users with low channel gains may be more vulnerable to threats like session hijacking, eavesdropping, and identity theft. Additionally, poor SIC might raise significant problems when implementing NOMA in real-world 5G networks. Therefore, better SIC schemes can address the imperfect SIC difficulties and significantly boost the performance of the NOMA system.

### 5.12. Security Issues

In NOMA, multiple user messages are superimposed in a single resource block (RB). The SIC receiver is used to decode the overlaid signals at the receiver. In the SIC receiver, the strong users are allocated with lower powers, and weak users are with strong powers for channel fairness. At the receiver, strong users have to subtract the low power signals to get their signals. But it causes some security issues, which have to be considered in NOMA. These security issues can be overcome using encryption and decryption schemes at the sender and receiver. This process increases the latency and processing requirements. But the basic idea of NOMA in 5G is to decrease the latency and to improve the data rates, spectral efficiency, and bandwidth. The NOMA implementation causes security issues such as the implementation of SIC, transmitted powers and outage probability. These security issues mainly occur in the physical layer. To overcome these security issues, optimal and analytical solutions must be thoroughly investigated.

## 6. Future Research Directions

We will discuss and outline potential future research directions here. Future research should focus on making NOMA technology smarter, more versatile, and easier to implement. The following are some directions that research into deep learning’s application to NOMA systems may go in the future.

### 6.1. Stability and Power Efficiency

When there is a temporary blockage, DL can adaptively reconfigure traffic among integrated access and backhaul (IAB) nodes to keep the link stable. In 5G mobile, preserving battery life is a top priority. Power can be saved by minimizing processing demands at the receiver. The potential of DL in the realm of power efficiency is worth investigating.

### 6.2. Channel Atatistics

When it comes to communication systems based on deep learning, the most significant challenge is creating a neural network. Using channel statistics, deep learning models with memory, like LSTM and RNN, may be used to forecast the channel’s behavior. Because of the time savings, this data can be utilized in place of real-time CSI.

### 6.3. Novel Algorithms

In addition to DL-based algorithms, unsupervised learning algorithms can address user clustering [44]. After learning the inherent frameworks and correlation between users, the expectation-maximization method can be adopted for user clustering in fixed and dynamic environments. NOMA can decrease the aggregation latency in FL model updating by ensuring multiple access in the same channel. In addition, based on well-trained DRL and DL, transfer learning can also be implemented for updating the partial/full neural network parameters with limited time and data.

### 6.4. Security

The research directions in cybersecurity shall focus on developing deep learning models with dynamic and adaptable characteristics to counter the rigorously growing attack scenarios and preparing up-to-date datasets characterizing real-world application data frames with balanced classes to validate these models as a possible solution to practical network attacks.

### 6.5. Receiver Complexity in SIC Implementation

In NOMA, the users are multiplexed at the transmitter side using pairing techniques and decoded at the receiver using the SIC receiver. As the number of users increases, the allocation of transmission powers to pair the users is very complex. At the same time, decoding the strong user to the next strong user and up to the weak user is a very difficult task at the receiver end. This process increases the latency and interference with the increased number of multiplexed users. Therefore, reducing the latency and interference and providing an efficient and dynamic SIC receiver for the NOMA system is a future challenge.

### 6.6. Multi-Cell NOMA System

In NOMA systems, most researchers considered a single cell, and very few addressed the multi-cell concept because the multi-cell system causes inter-cell interference. The interference will affect the performance of the weak users at the cell edge. Therefore, to solve the interference issues along with the pairing and decoding issues, a small cell concept of 5G is to be included in the multi-cell NOMA system. This is the future challenge in the multi-cell NOMA systems to be addressed.

### 6.7. Mobility in NOMA

NOMA is used to enable the 5G and beyond 5G technologies. In IoT, V2V and M2M communications mobility are the key parameters. Most of the research in NOMA systems is based on static systems. The power allocation, pairing and SIC receiver algorithms are proposed based on the static behavior of the users. But dynamic power allocation, pairing and SIC algorithms are required in future communications. As the user is moving from one location to another location, then the channel gains vary with respect to the user’s location. Therefore, proposing dynamic algorithms for NOMA systems is one of the future research areas.

### 6.8. CSI in NOMA

Most of the research is carried out by assuming a perfect CSI in NOMA systems, but less number of users are considered imperfect CSI. The CSI plays an important role in user pairing and decoding the users with SIC receivers in the NOMA system. To increase the system performance and spectrum efficiency of a dynamic system, the estimation of CSI is very important. For a dynamic channel, the estimation of CSI using machine learning and game theory algorithms is one of the important future research directions in NOMA systems.

## 7. Conclusions

This article surveys the existing studies and research contributions of deep learning-based NOMA systems. The use of DL methods indicates that DL has a high impact on solving complex communication challenges of NOMA systems due to its powerful learning ability. We have shown, without a doubt, that the implementation of DL methods in NOMA is paramount to enhancing the performance of NOMA-based wireless communication systems. In this context, we provide a detailed discussion about related works and key performance indicators. This article comprehensively discusses the extensive scope of DL-based NOMA systems with the integration of several emerging technologies such as MEC, IRS, SWIPT, OFDM and MIMO etc. In addition, we explore the diverse DL-based NOMA systems considered by recent works for power allocation, CSI, SIC, channel estimation, user fairness, impulse noise, transceiver design etc. In this article, we briefly explain the benefits and shortcomings of incorporating DL in NOMA systems. Also, we highlight and summarize the potential challenges and issues. We also focus on future research directions for ameliorating the performance of the DL algorithms for NOMA systems. The findings of our study indicate that the experimental evolution of DL-based solutions, better algorithms, making appropriate decision, and integration of emerging technologies can achieve low latency, less complexity, massive connectivity, higher capacity, higher spectral and energy efficiency, optimal resource allocation and better performance. We firmly believe that this article will capture the high interest of both academic and industrial fraternities to investigate the potential of DL-based approaches in the next general wireless communication systems. 

## Data Availability

The authors declare that they have no known competing financial interest or personal relationship that could have appeared to influence the work reported in this paper.
